# Lessons learnt from coordinating emergency health response during humanitarian crises: a case study of implementation of the health cluster in northern Uganda

**DOI:** 10.1186/1752-1505-9-1

**Published:** 2015-01-07

**Authors:** Olushayo Olu, Abdulmumini Usman, Solomon Woldetsadik, Dick Chamla, Oladapo Walker

**Affiliations:** World Health Organization (WHO) Inter country Support Team for Eastern and Southern Africa, Belvedere, PO Box BE 773, Harare, Zimbabwe; WHO, Asmara, Eritrea; WHO, Kampala, Uganda; UNICEF, New York, USA; Ibadan, Nigeria

**Keywords:** Emergency, Health, Response, Coordination

## Abstract

**Background:**

Between the late 1980s and 2000s, Northern Uganda experienced over twenty years of armed conflict between the Government of Uganda and Lord’s Resistance Army. The resulting humanitarian crisis led to displacement of a large percentage of the population and disruption of the health care system of the area. To better coordinate the emergency health response to the crisis, the humanitarian cluster approach was rolled out in Uganda in October 2005. The health, nutrition and HIV/AIDS cluster became fully operational at the national level and in all the conflict affected districts of Acholi and Lango in April 2006. It was phased out in 2011 following the return of the internally displaced persons to their original homelands.

**Conclusions:**

The implementation of the health cluster approach in the northern Uganda and other humanitarian crises in Africa highlights a few issues which are important for strengthening health coordination in similar settings. While health clusters are often welcome during humanitarian crises because they have the possibility to improve health coordination, their potential to create an additional layer of bureaucracy into already complex and bureaucratic humanitarian response architecture is a real concern. Although anecdotal evidence has showed that implementation of the humanitarian reforms and the roll out of the cluster approach did improve humanitarian response in northern Uganda; it is critical to establish a mechanism for measuring the direct impact of health clusters on improving health outcomes, and in reducing morbidity and mortality during humanitarian crisis. Successful implementation of health clusters requires availability of other components of the humanitarian reforms such as predictable funding, strong humanitarian coordination system and strong partnerships. Importantly, successful health clusters require political commitment of national humanitarian and government stakeholders.

**Recommendations:**

Although leaving health coordination entirely to governments (in crises where they exist) may result in political interference and ineffectiveness of the aid response efforts, the role of government in health coordination cannot be overemphasized. Health clusters must respond to the rapidly changing humanitarian environment and the changing needs of populations affected by humanitarian crises as they evolve from emergency towards transition, early recovery and development.

## Background

Disasters are common phenomena which disrupt socio-economic development and negatively impact on the health and nutrition status of the World’s population. According to the Centre for Research on the Epidemiology of Disaster (CRED), a yearly average of 392 natural disasters was recorded globally between 2000 and 2008 [1]. In 2012, 357 disasters affecting 124.5 million persons were reported globally representing an increase of 2.3% over the 2011 figures [2]. Africa bears the major burden of disasters whether natural or man-made; of the 30 largest complex emergencies and epidemic outbreaks between 1995 and 2004, 17 and 25 respectively occurred in Africa [3]. The continent is equally affected by armed conflicts; from the 1960s to 2008, about 24 sub-Saharan countries (more than half) of Africa experienced armed conflict [4]. Furthermore, the World Bank estimates that one in every three African is directly or indirectly affected by conflicts which may delay the attainment of international development goals [5].

Effective emergency response to disasters is often constrained by weak coordination. Since the early 1970’s and 1980s, the number of complex emergencies which require special coordination bodies have increased [6], as has the number of humanitarian partners [7] thereby complicating humanitarian coordination. The presence of many humanitarian partners usually results in a scramble for relevance and scarce resources such as human, financial and logistics which increases the cost and reduces the effectiveness of emergency response [4]. This is often further complicated by limited humanitarian space due to insecurity, lack of physical access to affected populations and poor political commitment of warring parties or national governments [8]. In Africa, high levels of humanitarian needs persist [9], and if the humanitarian system is to make impact in Africa, it must be more systematic in the way it approaches humanitarian crises [8]. This includes taking focused and coordinated steps to identify the level of need, to build, re-establish and employ indigenous early warning, preparedness and response capacity and to commit to funding such initiatives in an equitable and predictable way [10].

An independent review of the global humanitarian system was commissioned in 2005 to better understand and correct the deficiencies in global humanitarian response [11]. The report of this review formed the basis of a report by the United Nations (UN) Secretary-General to the General Assembly on strengthening of the coordination of UN humanitarian assistance. The report examined some of the key humanitarian developments and challenges, particularly capacity gaps experienced in both complex emergencies and disasters and highlighted the large scale of deaths, displacements, injuries and destructions caused by the large scale conflicts and mega-natural disasters including the Indian Ocean Tsunami [12].

In considering the report of the Secretary-General, the 60th session of the UN General Assembly adopted resolution A/RES/60/124 on the strengthening of the coordination of emergency humanitarian assistance of the UN [13]. This resolution formed the main basis for an International Humanitarian Reform Programme with three main pillars namely strengthening of the humanitarian coordination system, strengthening humanitarian response capacity especially in areas with gaps through the roll-out of a humanitarian cluster approach and establishment of an emergency response funding mechanism which would ensure timely and adequate financing for humanitarian action. A fourth pillar, “building more effective partnerships between UN and non-UN humanitarian actors” was also proposed to serve as a foundation for the first three pillars [14]. The cluster approach refers to a system through which humanitarian partners (both UN and non-UN) are grouped together (based on their mandate and comparative advantage) to strengthen coordination of the key sectors in humanitarian response such as health, nutrition, water and sanitation [15]. Clusters are designated by the global Inter Agency Standing Committee (IASC) based on the recommendations of Humanitarian Country Teams (HCT) and gap areas in humanitarian response.

To implement this international humanitarian reform programme, nine clusters were designated at the global level. These clusters included the Health Cluster, whose main objectives were to provide health leadership for emergency preparedness, response and recovery; prevent and reduce emergency related morbidity and mortality; ensure evidence based health actions, gap filling and sound coordination; and enhance accountability, predictability and effectiveness of humanitarian health action [16]. Uganda, Liberia, Somalia and the Democratic Republic of Congo (DRC), countries experiencing chronic emergencies at the time, were selected as pilot countries in Africa.

In this article, we review the key issues in emergency health response coordination using the experiences, successes, challenges and lessons learned from the implementation of the Health, Nutrition and HIV/AIDS Cluster (HNHAC) in Uganda. Based on the lessons learned from Uganda and other similar countries that have implemented the cluster approach, we propose a few recommendations which can be used to improve health coordination during both acute and chronic humanitarian crises.

## Methodology

This article is a retrospective analysis of the humanitarian response to the northern Uganda crisis with particular emphasis on the operations of the HNHAC in improving health response. The main methodology used for the analysis was desk reviews of various documents on the northern Uganda crisis. Information for the introductory section was obtained from the humanitarian response review and reform documents as well as reports of joint assessments and various surveys. The issues, challenges and lessons learnt were obtained from a review of the minutes of cluster meetings, joint project and cluster evaluation reports, annual reports and monthly bulletins of the HNHAC. Key informant interviews were also held with selected HNHAC members and also with key Ministry of Health (MOH) officials to further validate the findings of the desk review.

Two out of the five authors participated actively (as coordinator and chairpersons) in many of the cluster meetings and used this as opportunities for “participant observation” of the cluster dynamics. A third author attended selected cluster meetings as an independent observer and also provided further insights into the cluster dynamics and understanding of the principles of the humanitarian reforms and cluster approach by cluster members.

## The context: northern Uganda

Between the 1980s and 2000s, northern Uganda experienced over twenty years of armed conflict between the Government of Uganda (GoU) and Lord’s Resistance Army (LRA). The resulting humanitarian crisis led to displacement of a large percentage of the population and disruption of the health care system of the area. At the height of the conflict, over 90% of the population of Acholi sub-region (which comprise Gulu, Kitgum, Pader and now Nwoya and Amuru districts) and a lesser percentage of the population of Lango sub-region (comprising Apac, Lira and now Oyam, Amolatar and Dokolo districts) were displaced into Internally Displaced Persons (IDP) camps [17]. Living conditions in the IDP camps were extremely poor. According to the results of a mortality survey conducted in 2005, overcrowding, limited access to social services such as health, water and sanitation, violence and insecurity in the camps resulted in high Crude Mortality Rates (CMRs) of 1.54 and Under Five Mortality Rate (U5MR) of 3.18/10,000/day among the IDPs [18]. The high CMR was largely due to malaria/fever, HIV/AIDS and violence inflicted by others (including rape and gender based violence) in that order of importance. Among children under five years, malaria/fever and malnutrition/diarrhoea were the main causes of death. The findings of this survey were a major catalyst which attracted global attention to the northern Uganda crisis and was one of the reasons for rolling out the cluster approach in the country.

Since the landmark Cessation of Hostilities Agreement (CHA) between GoU and LRA was signed in August 2006, northern Uganda has witnessed significant improvements in the security and peace situation. This stability resulted in spontaneous return of the IDPs to their original homelands. Currently, more than 98% of the IDP populations in both Acholi and Lango sub-region have returned to their original homelands or have resettled in new locations [19].

## Emergency response to the northern Uganda crisis

The humanitarian response to the northern Ugandan crisis was done through a combination of project and budget support approaches. A significant percentage of external funding (70% to 80%) was channelled through the international humanitarian response mechanism which comprise of UN agencies, national and international Non-Governmental Organizations (NGOs) and Civil Society Organizations (CSOs) to directly support projects aimed at alleviating the humanitarian consequences of the crisis in the IDP camps. Almost all the funding from government was provided as budget support to the administration of the affected districts to support humanitarian as well as developmental activities. Within the health sector, the MOH had the overall oversight for implementation of the emergency health response activities while the District Health Management Teams (DHMT) were directly responsible for the implementation and coordination of the response activities at the district level. The dual approach resulted in proliferation of humanitarian partners, which necessitated the establishment of an effective humanitarian coordination system.

## Coordination of health services delivery

**Figure 1:**
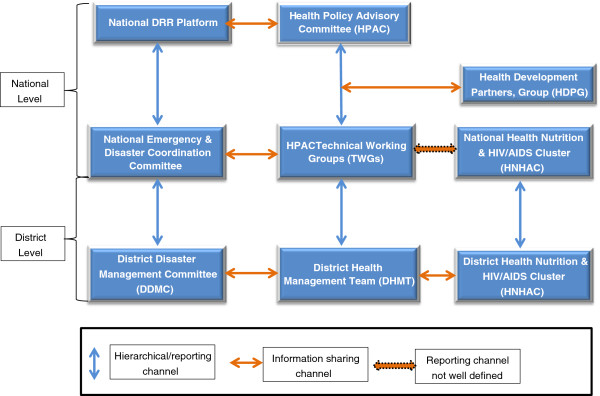
**Schematic diagram of the health and disaster coordination mechanism in Uganda (2006 – 2011).**

Delivery of health care in Uganda rests on the Health Sector Investment Plan III (HSIP III) which is implemented using a Sector Wide Approach (SWAP). Under this approach, a Health Policy Advisory Committee (HPAC) chaired by the Permanent Secretary (PS) of the MOH is the overall operational, advisory and coordination body within the health sector. HPAC has several Technical Working Groups (TWGs) where technical issues are discussed in-depth and viable options are agreed upon before presentation to HPAC for final decision making. The development partners (comprising of donors and other stakeholders) who provide support to the health sector of Uganda also have a forum known as Health Development Partners Group (HDPG) where issues of common interest are discussed and consensus reached before engaging MOH via HPAC. Agreed positions are presented to HPAC by the chair of the HDPG. The above policy making and coordination applies mainly to developmental issues within the health system of the country. The Office of the Prime Minister (OPM) coordinates disaster and emergency related issues. The Disaster Risk Reduction (DRR) platform which comprises of relevant government Ministries, UN agencies, national and international NGO and is chaired by the OPM serves as the overall coordination mechanism for all disaster and emergency responses. The DRR platform holds monthly meetings and spearheads the development and implementation of the policy for disaster management which was approved by parliament recently. The platform has several technical working groups which comprise of government line Ministries or sectors. However, the participation of the sectors in the DRR platform is limited, resources are inadequate and emergency preparedness interventions limited (Figure 1).

## Coordination of the emergency health response to the northern Ugandan crisis

The cluster approach was rolled out in Uganda in October 2005 with the designation of five pilot clusters including the HNHAC. The introduction of the approach was done with little or no consultation with relevant national authorities, UN agencies and NGOs present in the country [20]. The cluster became fully operational at the national level and in all the conflict-affected districts of Acholi and Lango sub-regions in April 2006 with the designation of two working groups namely health and nutrition and HIV/AIDS. The cluster core commitments and objectives included effective coordination of the emergency health, nutrition and HIV/AIDS response in northern Uganda and other conflict-affected areas, joint assessments, monitoring and reporting of the health, nutrition and HIV/AIDS situation, capacity building, application of standards and resource mobilization among others. Unlike some of the other clusters, health cluster coordination functions were combined with the emergency and humanitarian action functions of the cluster lead agency, the World Health Organization (WHO).

There was minimal functional or technical interaction between the HNHAC and the national health sector coordination mechanism (HPAC) due to a number of reasons. Firstly, none of the HPAC working groups had direct responsibility for humanitarian response; secondly, due to inadequate staffing, it was not possible for the MOH to second a fulltime staff member to work with the HNHAC; thirdly, as result of the minimal consultation with the national authorities during the introduction of the cluster approach to the country, many of the key MOH officials were not aware of its existence and in cases where they were, had very limited understanding about how it worked. In many instances, the cluster approach was equated to the SWAP which the GoU was already implementing thus resulting in further confusion about the cluster approach and compounding the belief that it was duplicating government efforts. However, at the district level, government participation in HNHAC activities was much better largely due to the fact they (affected districts) were cashed strapped and needed the supplementary funding brought by the cluster approach.

In its five years of existence, the health cluster implemented several key activities which contributed to better coordination of the emergency health response in the conflict affected areas. These activities included among others, monthly (and sometimes weekly) health cluster meetings at the national and district levels, conduction of several surveys including the health services availability mapping surveys (conducted in Acholi sub-region in 2006 and in Lango sub-region in 2007), health and human rights survey (conducted in Acholi sub-region in 2007) and gender based violence risk assessment (done in Kitgum district in 2007); these surveys provided information for evidence-based planning. In addition, quarterly cluster bulletins, monthly cluster reports and biannual mapping of cluster partners were produced and disseminated widely to improve information sharing while several cluster training workshops were held. From 2006 to 2009, the cluster supported the planning, implementation, supervision, monitoring and evaluation of a joint inter agency emergency health, nutrition and HIV/AIDS response programme in the IDP camps of northern Uganda. Between 2007 and 2010, the health cluster also supported the MOH to develop a health sector recovery strategy and plan aimed at rebuilding (back better) the health system of the conflict affected districts of northern Uganda.

**Figure 2:**
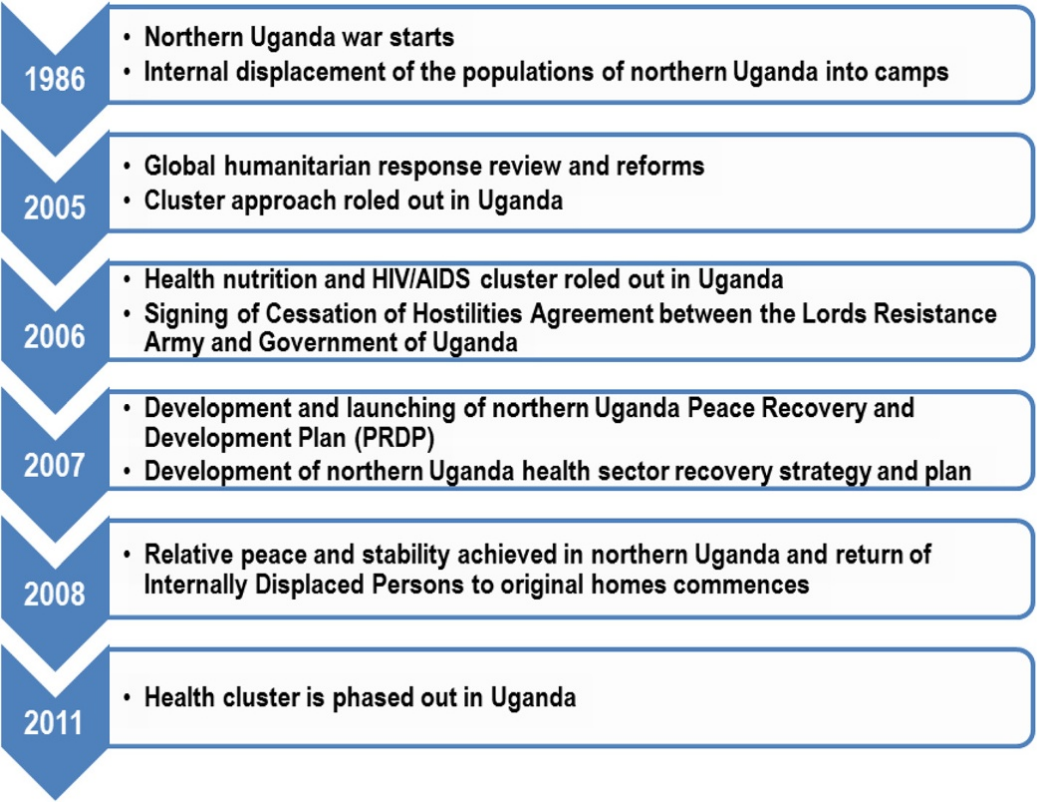
**Timeline of key events in northern Uganda.**

With the prevailing peace in northern Uganda and the return of majority of the IDPs to their original homelands or resettlement in new locations, the humanitarian situation in these parts of the country has improved. HNHAC was gradually phased out from 2009 to 2011 and its activities were merged into the already existing health sector coordination mechanisms covering all the conflict affected districts of northern Uganda (Figure 2).

## Lessons learnt from implementation of the health cluster

The implementation of the health cluster approach in the northern Ugandan and other humanitarian crises in Africa highlights a few critical issues which are important for strengthening health coordination in similar settings. While health clusters are often welcome during humanitarian crises as they have the possibility to improve health coordination, their potential to create an additional layer of bureaucracy into already complex and bureaucratic humanitarian response architecture is a real concern. The use of a top-down approach for health cluster activation (as was the case in Uganda) often creates poor understanding of the cluster approach systems which ultimately results in poor ownership by national governments and stakeholders. Under such circumstances, reconciling the differences in the mandates and agenda of MOH and health cluster partners becomes a daunting challenge. The Uganda experience showed that reconciling such differences was found to be much easier to achieve during acute crises as compared to chronic ones. This is perhaps due to the national and international attention which acute emergencies often generate and the attending political pressure on government and health cluster partners to quickly bring such crises under control. Coordination across sectors and addressing cross cutting issues still remains a serious challenge. Health sector response during disaster requires strong collaboration with the partners working in the other sectors especially, water, sanitation, camp management, food and agriculture, and other relevant sectors. Given the limited capacity available, both locally and globally, and the urgency for instituting lifesaving interventions for the population at risk this additional responsibility of reaching out to other sectors can be a daunting challenge.

In many countries experiencing humanitarian crises, the terms “health sector” and “health cluster” are often used interchangeably. However, the Ugandan experience did demonstrate the importance of differentiating between these two terminologies (and their roles and responsibility in humanitarian crises) especially in countries implementing SWAP. Understanding these differences has far-reaching implications for ensuring a smooth transition from emergency health response to recovery and development of the health system [21]. While health clusters’ functions are specific and limited to coordination of emergency health response during humanitarian crises, health sectors have wider and longer-term (more or less permanent) coordination responsibilities which include health system development, policy making, overall coordination of health service delivery (including emergency health response), recovery and management of health disaster risks. This highlights the need to ensure very close collaboration between health clusters and sectors during all the phases of an emergency.

The role of health clusters in transition, health system recovery and post-emergency development is unclear. Although in Uganda the health cluster supported the DHMT and MOH to develop a health recovery strategic document and plan of action, the limited interaction and collaboration between the health sector and cluster impeded the timely implementation of the plan for three main reasons. Firstly, some health sector partners felt that there was no need for a health system recovery strategy and plan as health system recovery was already addressed in national health strategies and plans albeit very scantily. Secondly, the health sector partners had inadequate knowledge about the key issues in health transition from emergency to recovery and development (and the need for a systematic approach to recovering health systems post-conflict/disaster) while on the other hand most health cluster partners (who are mainly emergency oriented) lacked a clear understanding of the approaches to the health system recovery and development. Thirdly, the lack of appropriate guidelines for managing health system recovery also significantly contributed to the delayed implementation of the recovery programme.

The use of fulltime versus part-time health cluster coordinator (who have other programme responsibilities) remains a contentious issue. While dedicated cluster coordinators are preferred, their sustainability over time is a major challenge. In Uganda, the health cluster was managed by a part-time (double hatted) cluster coordinator, an arrangement which had its pros and cons. While the cluster coordinator had direct control of the cluster lead agency’s resources such as funds, emergency field staff and logistics support system and could deploy these to facilitate timely cluster response to emergency situations and effectively fulfil the cluster lead agency provider of last resort functions, it was often difficult to avoid conflict of interest between the two roles. To the best of our knowledge, to date Uganda is the only country where health, nutrition and HIV/AIDS were combined into one cluster. These had important pros and cons; the combination ensured that HIV/AIDS, a cross cutting issue was given enough attention within the cluster. The HIV/AIDS working group of the cluster had membership from all sectors which facilitated the mainstreaming of the health aspects of HIV/AIDS into the other sectors. The inclusion of nutrition in the health cluster ensured that the inter linkages between health and nutrition were well explored and properly addressed. However, the difference in leadership of the health and nutrition clusters at the global and country levels resulted in inadequate technical support from the global nutrition cluster to the HNHAC.

The cluster approach employs voluntary participation among the various stakeholders operating in an area of disaster. Though in principle NGOs and UN agencies are all accountable both to the beneficiaries and the donors, in practice there is no binding rule to ensure proper coordination and bring all stakeholders to rally behind a comprehensive plan that is developed with full participation of all, including the government, beneficiaries, NGOs and UN agencies. In spite of this drawback, experience from Uganda shows that provided that there is good leadership at all levels, it is possible to bring most partners including the government to work together for the betterment of the life of beneficiaries. The importance of donor cohesion and support in this regard cannot be overemphasized [22, 23]. The lessons learnt from implementation of the joint inter agency emergency health, nutrition and HIV/AIDS response programme in northern Uganda which was jointly funded by Department for International Development (DFID) of the United Kingdom and the Swedish Development Agency (Sida) using the HNHAC platform showed that donors have important roles to play in bringing humanitarian partners to the negotiation table. Such joint programmes have the ability to help health cluster partners to easily define their mandates and comparative advantages which will in turn facilitate transparent allocation of tasks and responsibilities, ensure accountability (to beneficiaries, partners and donors) and ultimately reduce duplication of efforts.

To address the inherent challenges of weak humanitarian leadership, lack of clear accountability framework in humanitarian response and the overly process driven approach of humanitarian clusters, the IASC Principals agreed on a set of actions (called the transformative agenda) in late 2011/early 2012 [24]. The agenda aims to improve humanitarian leadership through deployment of experienced humanitarian leaders at the beginning of humanitarian crises, strengthen joint strategic planning which defines the shared outcomes that should be collectively achieved by all humanitarian partners, improve accountability of partners to beneficiaries and to ensure use of context specific coordination mechanisms during humanitarian crises. If well implemented, this agenda would significantly contribute to successful implementation of the humanitarian reforms especially roll out of humanitarian health clusters.

The phasing out of HNHAC proved to be an onerous task due to a number of reasons. HPAC which was supposed to take over its function was already overloaded and had limited capacity to take on additional responsibilities. Although it (HPAC), is the highest coordination and decision making body in the MOH and has several TWGs, some of which could take over the health cluster responsibilities, there were concerns that these TWGs already had several other agenda and as a result health system recovery and development in northern Uganda would not receive enough attention. Perhaps the most plausible reason for the poor integration of the health cluster into the HPAC structure is the poor collaboration between both bodies ab-initio.

## Study limitations

The findings and conclusions of this study may have been biased by the active involvement of two of the authors as cluster coordinators and chairpersons of the cluster meetings at various times. A number of steps were taken to mitigate this bias; key informant interviews with cluster members were used to validate the findings of the authors’ participant observations. Furthermore, findings of the independent cluster evaluations were also used to corroborate the observations of the authors. Participation of one of the authors as an independent observer in some of the cluster meetings also provided further independent information which were used to cross check the findings and conclusions of this study.

## Conclusions and recommendations

Although anecdotal evidence have shown that implementation of the humanitarian reforms and roll out of the cluster approach did improve humanitarian response in northern Uganda [25]; it is important to establish a mechanism for measuring the direct impact of health clusters on improving health outcomes, reducing morbidity and mortality during humanitarian crisis. Many critical issues which are discussed in this article need to be further defined in order to consolidate the achievements made so far in the implementation of the health cluster in the African region and at the global level. Successful implementation of health clusters requires availability of other components of the humanitarian reforms namely predictable funding, a strong humanitarian coordination system, strong partnerships with MOHs and national health partners and implementation of the transformative agenda. Importantly, successful health clusters require top political commitment of national humanitarian and government stakeholders.

Drawing from experiences and lessons learned from the health cluster implementation in Uganda and coordination of humanitarian crises in Afghanistan, Mozambique, Rwanda and Pakistan [26] we propose a few suggestions to improve health cluster roll-out and implementation during future emergencies:

1. Coordination is a means to an end and not an end in itself [27], hence health clusters and their partners must ensure that they keep the end in focus at all times by creating a balance between time allocated to coordination activities including meetings and the actual task of delivering services to affected populations.
2. It is important to ensure that health cluster partners see and reap the benefits of participating in the health cluster coordination mechanism. Health clusters must create demand for coordination by demonstrating that their benefits offset their disadvantages. The clusters must do business differently from the coordination systems that existed before them (if any) and ensure that it effectively performs it roles while at the same time ensuring that it does not create another layer of bureaucracy for programme implementation.
3. Health clusters should invest in ensuring that its partners understand and respect the mandates of each other and ensure that decision making within the cluster is transparent, evidence based and is by consensus.
4. Although leaving health coordination entirely to governments (in crises where they exist) may result in political interference and ineffectiveness of the aid response efforts, the role of governments in health coordination cannot be overemphasized. For this reason, it is critical for health clusters to build on and safeguard existing government health coordination mechanisms to ensure sustainability. In this regard, health cluster lead agencies and partners should ensure MOH leadership in the conceptualization, planning, implementation, monitoring and evaluation of health clusters. Furthermore, health cluster engagement of national MOHs would facilitate standard setting and regulation since the government has the primary mandate for doing this.
5. Donors have a strong role to play in successful roll out and implementation of health clusters and the health cluster lead agency and partners should ensure that they are involved at every stage of the cluster implementation.
6. The decision to deploy a full or part-time cluster coordinator should be guided by the context, the prevailing humanitarian situation and availability of predictable and sustainable funding to the cluster lead organization which further underscores the importance of donor participation in health cluster activities.
7. Health clusters must respond to the rapidly changing humanitarian environment and the changing needs of populations affected by humanitarian crises as the crises evolves from emergency towards transition, early recovery and development.
8. The role of health clusters in health services delivery during the transition, early recovery and development phases should be clearly defined using durable, sustainable and context-specific models. In this regard, building the capacity of health cluster partners on post conflict/disaster health system recovery is key.
9. Establishment of new health clusters should be done within the framework of the transformative agenda. This would foster stronger leadership, development of joint and mutually agreeable strategic plans and most importantly accountability of its members to both beneficiaries and donors.
10. Health clusters must develop and negotiate clear exit strategies right from their inception and gradually work toward implementing these strategies as a humanitarian crisis progresses. They (health clusters) should focus on gradually building the capacity of relevant government partners or mechanisms (through training, technical backstopping, monitoring and evaluation) to ensure that they can take over full coordination of emergency response and early recovery efforts as soon as practicable during a humanitarian crisis.

## Authors’ information

OO was the HNHAC coordinator and head of WHO emergency and humanitarian action programme in Uganda from 2005 to 2009. He was health cluster coordinator for Zimbabwe from February to July 2009 and is currently the Outbreak and Disaster Management (ODM) focal point of WHO for eastern and southern Africa. In this position, he has oversight for the health clusters in the sub-region including Uganda.

AU was the WHO Technical Officer in charge of response and recovery operations in the African region. In that position, he had oversight for all the health clusters in the region including Uganda. He participated in several technical support missions to the health clusters. He is currently the WHO Representative to Eritrea.

SW was the HNHAC coordinator and head of WHO emergency and humanitarian action programme in Uganda from 2009 to 2011.

DC was a Technical Officer in WHO Uganda from 2005 to 2008. He provided technical support to conduct the mortality survey and several other surveys in northern Uganda and also to roll out the health cluster in the country. He continues to provide oversight to the country in his current position in UNICEF headquarters.

OW was WHO Representative to Uganda at the height of the crisis. He provided the overall supervision for WHO’s response to the northern Uganda crisis and was also in the forefront of rolling out the health cluster.

## Abbreviations

CHA: Cessation of Hostility Agreement
CMR: Crude Mortality Rate
CRED: Centre for Research on the Epidemiology of Disaster
CSO: Civil Society Organization
DFID: Department for International Development
DHMT: District Health Management Team
DRR: Disaster Risk Reduction
GBV: Gender Based Violence
GoU: Government of Uganda
HDPG: Health Development Partners’ Group
HNHAC: Health, Nutrition and HIV/AIDS Cluster
HPAC: Health Policy Advisory Committee
HSIP: Health Sector Investment Plan
IASC: Inter Agency Standing Committee
IDP: Internally Displaced Person
LRA: Lord’s Resistance Army
MOH: Ministry of Health
NGO: Non-Governmental Organization
ODM: Outbreak and Disaster Management (programme of WHO)
PS: Permanent Secretary
Sida: Swedish International Development Cooperation Agency
SWAP: Sector Wide Approach
TWG: Technical Working Group (of HPAC)
UN: United Nations
WHO: World Health Organization.
